# Evaluation of the Antigen-Experienced B-Cell Receptor Repertoire in Healthy Children and Adults

**DOI:** 10.3389/fimmu.2016.00410

**Published:** 2016-10-17

**Authors:** Hanna IJspeert, Pauline A. van Schouwenburg, David van Zessen, Ingrid Pico-Knijnenburg, Gertjan J. Driessen, Andrew P. Stubbs, Mirjam van der Burg

**Affiliations:** ^1^Department of Immunology, Erasmus MC, University Medical Center Rotterdam, Rotterdam, Netherlands; ^2^Department of Bioinformatics, Erasmus MC, University Medical Center Rotterdam, Rotterdam, Netherlands; ^3^Department of Pediatrics, Erasmus MC-Sophia Children’s Hospital, University Medical Center Rotterdam, Rotterdam, Netherlands

**Keywords:** somatic hypermutation, class switch recombination, next generation sequencing, reference data set, repertoire, immunoglobulins

## Abstract

Upon antigen recognition via their B cell receptor (BR), B cells migrate to the germinal center where they undergo somatic hypermutation (SHM) to increase their affinity for the antigen, and class switch recombination (CSR) to change the effector function of the secreted antibodies. These steps are essential to create an antigen-experienced BR repertoire that efficiently protects the body against pathogens. At the same time, the BR repertoire should be selected to protect against responses to self-antigen or harmless antigens. Insights into the processes of SHM, selection, and CSR can be obtained by studying the antigen-experienced BR repertoire. Currently, a large reference data set of healthy children and adults, which ranges from neonates to the elderly, is not available. In this study, we analyzed the antigen-experienced repertoire of 38 healthy donors (HD), ranging from cord blood to 74 years old, by sequencing IGA and IGG transcripts using next generation sequencing. This resulted in a large, freely available reference data set containing 412,890 IGA and IGG transcripts. We used this data set to study mutation levels, SHM patterns, antigenic selection, and CSR from birth to elderly HD. Only small differences were observed in SHM patterns, while the mutation levels increase in early childhood and stabilize at 6 years of age at around 7%. Furthermore, comparison of the antigen-experienced repertoire with sequences from the naive immune repertoire showed that features associated with autoimmunity such as long CDR3 length and IGHV4-34 usage are reduced in the antigen-experienced repertoire. Moreover, IGA2 and IGG2 usage was increased in HD in higher age categories, while IGG1 usage was decreased. In addition, we studied clonal relationship in the different samples. Clonally related sequences were found with different subclasses. Interestingly, we found transcripts with the same CDR1–CDR3 sequence, but different subclasses. Together, these data suggest that a single antigen can provoke a B-cell response with BR of different subclasses and that, during the course of an immune response, some B cells change their isotype without acquiring additional SHM or can directly switch to different isotypes.

## Introduction

During B-cell development in the bone marrow, each B cell creates its own unique B cell receptor (BR) in a process called V(D)J recombination. The heavy chain encoding IGH locus is generated by recombining one of the 38–46 functional variable (V), 25 diversity (D), and 6 joining (J) genes on the antigen receptor loci. Small deletions and the addition of non-templated (N-) and palindromic (P-) nucleotides during this process enables B cells to make an even larger variation of BRs. The light chain can be either encoded by the IGK or IGL locus and is generated by VJ recombination. Before the immature B cells leave the bone marrow, the BR is tested for autoreactivity. Autoreactive B cells either go into apoptosis, become anergic, replace their VH, or rearrange the kappa locus (i.e., receptor editing) to change their specificity.

The naive repertoire formed upon V(D)J recombination often encodes low affinity antibodies. Upon antigen recognition and T-cell activation, reactive B cells home to germinal centers where they can undergo two additional maturation steps: somatic hypermutation (SHM) and class switch recombination (CSR). During SHM, point mutations are introduced, which accumulate in the rearranged VDJ gene. Afterward, cells with affinity-increasing mutations get selected for survival, while cells carrying mutations that reduce affinity undergo apoptosis. CSR changes the constant region of the BR, thereby changing its effector function. SHM and CSR are both initiated by activation-induced cytidine deaminase (AID), which converts a cytidine (C) into a uracil (U) by deamination resulting in a U:guanine (G) mismatch. AID has been shown to preferentially target RGYW (R = Purine, Y = pyrimidine, W = A or T) and WRCY motifs for deamination (1, 2).

During SHM, a U:G mismatch can be processed by different error-prone DNA repair pathways (Figure 2A). First, the U can be recognized by members of the base excision repair (BER) machinery, which leads to replacement of the U by error-prone polymerases resulting in transition and transversion mutations at G/C bases (3, 4). Mutations at A/T bases can occur when the mismatch is recognized by the mismatch repair (MMR) machinery, whereby multiple bases around the mismatch are removed and replaced by error-prone polymerases. These mismatches are predominately introduced by polymerase (pol)η, which introduces errors at A/T pairs, specifically in WA/TW motives (5–11), however polζ (12, 13), polκ (14), polι (15), and more controversially polθ have also been associated with SHM (16–19). If the U:G mismatch is not repaired before the DNA is replicated, the U will be recognized as a thymine (T), resulting in a C to T transition mutation.

In the periphery, the repertoire can be divided in naive and antigen-experienced repertoire. The naive repertoire is formed upon V(D)J recombination, and potentially receptor editing, but is not selected by antigens. In contrast, the antigen-experienced repertoire is changed by SHM, CSR, and selection. Recently, we described the characteristics of the naive repertoire in healthy donors (HD) (20), but a large reference data set of the antigen-experienced BR repertoire in HD of all different ages (neonates to elderly) is missing. Small studies on SHM have shown low frequency of mutations in cord blood samples and infants younger than 2 months (21), and a rapid increase in the mutation frequency during the first 2 years of life (22). While no changes in the mutation frequency were observed in young adults compared with elderly (23). Another study investigated CSR in young adults and elderly and described increased IgA2 and IgG2 usage in elderly (24).

In this study, we investigated the antigen-experienced repertoire with the aim of studying how SHM, selection, and CSR are influenced by age. We describe the antigen-experienced repertoire of 38 HD from cord blood to the age of 74 years. We used next generation sequencing to generate this reference data set and analyzed the repertoire using an updated version of our IGGalaxy analysis pipeline (25).

## Materials and Methods

### Healthy Donors

Peripheral blood and cord blood samples were collected from 38 HD of different ages (Supplementary Table 1 in Data Sheet 2). From all donors, peripheral blood or cord blood was obtained with informed consent and according to the guidelines of the Medical Ethics Committees of the Erasmus MC.

### Repertoire Sequencing Using Next Generation Sequencing

PBMC’s were isolated from peripheral blood or cord blood samples using Ficoll separation. mRNA was isolated using the GenElute Mammalian total RNA miniprep kit from Sigma Aldrich (St. Louis, MO, USA). cDNA was created from 2 μg RNA using the Superscript II reverse transcriptase kit from Invitrogen (Paisley, UK) in a 40 μl reaction. IGH transcripts were amplified from 5 μl cDNA per reaction in a multiplex PCR using the forward VH1-6 FR1 (BIOMED-2) primers (26) and either the CgCH1 (27) or the IGHA (28) reverse primer. These PCR products were purified and sequenced using Roche 454 sequencing as previously described (29). In short, PCR products were purified by gel extraction (Qiagen, Valencia, CA, USA) and using Agencourt AMPure XP beads (Beckman Coulter, Fullerton, CA, USA). Subsequently, the concentration of the PCR product was measured using the Quant-it Picogreen dsDNA assay (Invitrogen, Carlsbad, CA, USA). The purified PCR products were sequenced on the 454 GS junior instrument using the Lib-A V2 kit according the manufacturer’s recommendations. Data processing was performed using the long-amplicon pipeline 1 from Roche.^1^ This pipeline is similar but more stringent to the standard Amplicon pipeline,^2^ resulting in the lowest throughput, but the highest quality filtering.

### Immune Repertoire Data Analysis

Sequences were demultiplexed based on their multiplex identifier sequence and 40 nucleotides trimmed from both sides to remove the primer sequence using the IGGalaxy tool (25). Fasta files were uploaded in IMGT/High-V-Quest (selection of IMGT reference directory set: F + ORF + in-frame P with all alleles, search for insertions and deletions: yes, parameters for IMGT/Junction Analysis: default) (30), and subsequently, the IMGT output files were analyzed in an extended version of our IGGalaxy tool (ARGalaxy) (25). Information on junction characteristics, CDR3 length and composition, CSR, the number of mutations, and SHM patterns were obtained. Only productive sequences, that were complete, without ambiguous bases, present twice or more, with a C subclass that could be defined, were included in the analysis as a single sequence. All information on the FR1 region was excluded from the analysis since the forward primers used to amplify the transcripts were located in FR1. The age, gender, and number of sequences after each filtering step are listed in Supplementary Table 1 in Data Sheet 2, and detailed information on the mutations used to calculate the SHM patterns (transition tables) are listed in Supplementary Table 2A (IGG) and 2B (IGA) in Data Sheet 2. In addition, the immunoglobulin analysis tool (IgAT) (31) was used to determine the percentage of antigen-selected sequences. To determine the selection strength of the CDR and FR regions, the BASELINe tool was used (selection statistics: focused, SHM targeting model: human Tri-nucleotide, custom boundaries: 25:26:38:55:65:104:-) (32, 33).

Clonal relation between sequences was determined using Change-O using the nucleotide hamming distance substitution model with a complete distance of maximal three. For clonal assignment, the first genes were used, and the distances were not normalized. In case of asymmetric distances, the minimal distance was used (34).

Lineage trees were created based on a minimal substitution model. The clonal evolution of the sequences was determined by identifying mutations that overlap between all sequences or groups of sequences. The tree structure was based on the model in which the least number of mutations was needed to create all different transcripts present in the clone, for details see Supplementary Table 4 in Data Sheet 2.

For the naive repertoire data, previously published data were used for analysis (20). In short, the demultiplex and trimmed fasta files were uploaded into IMGT/High-V-Quest and, subsequently, the IMGT output files were analyzed in an extended version of our IGGalaxy tool (ARGalaxy) (25, 30). Only productive sequences with a unique combination of their VH, JH, and amino acid sequenced of the CDR3 were used for the analysis.

### Data Availability

FASTQ files of the raw and filtered data of the naive B cell repertoire and IGG and IGA transcripts are available from the European Nucleotide Archive (ENA) project number PRJEB15348.

## Results

### Analysis of IGG and IGA Transcript in Peripheral Blood from Healthy Individuals

To study the antigen-experienced repertoire of HD of different ages, IGG and IGA transcripts from peripheral blood of 36 HD (age: 1–74 years; 18 males and 18 females), and two female cord blood samples were sequenced using next generation sequencing (Supplementary Table 1 in Data Sheet 2). We used Roche 454 sequencing because of its ability to handle the long IGG and IGA amplicons (700–800 bp).

For analysis, only transcripts assigned as functional by IMGT/High-V-Quest were selected (30). To exclude low quality reads, sequences containing uncalled “N” bases in the CDR1-CDR3 sequence were excluded and reads missing a CDR1, FR2, CDR2, or FR3 sequence were discarded. To allow analysis of SHM, an additional filtering strategy was developed, which allows better distinguishing between sequencing errors and true mutations. Yaari et al. proposed a strategy in which only unique reads of which the exact CDR1-CDR3 nucleotide sequence occurs two or more times are used for further analysis (35). HD in which this filtering resulted in less than 45 unique transcripts were excluded from further analysis (IGG *n* = 5 and IGA *n* = 1). This filtering resulted in a unique reference data set of 31,380 IGG and IGA transcripts derived from 38 individuals, which is deposited, so publically available for other studies.

### The Frequency of Mutations Increases during Early Childhood

**Figure 1:**
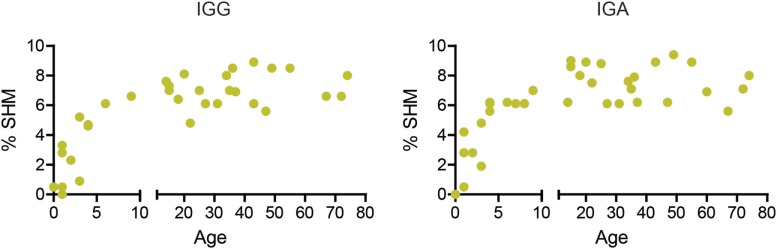
**The percentage of mutations increases in early childhood**. The median percentage of SHM in IGG and IGA transcripts increases with age in young children but stabilizes around 7% at 6 years of age.

During early childhood, the peripheral B-cell compartment changes significantly, with an increase in the absolute numbers of B cells and the percentage of memory B cells (36). However, SHM has not been studied extensively in children and adults. We found that the percentage of mutations in both IGA and IGG transcripts increased during early childhood and remained around 7% (range 5.6–9.4%) from the age of about 6 years onward (Figure 1).

### Targeting and Repair of SHM

**Figure 2:**
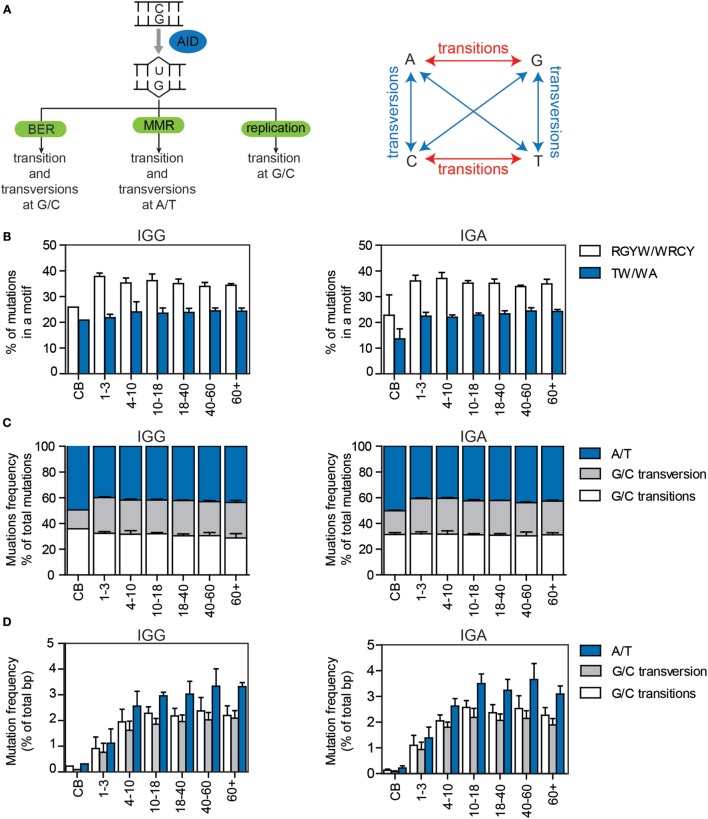

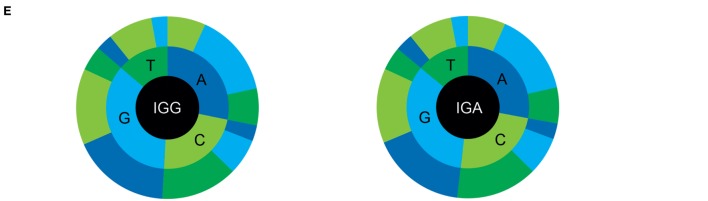
**The targeting and patterns of SHM in IGG and IGA transcripts of HD of different ages**. **(A)** Schematic overview of the different pathways by which AID-induced U:G mismatches can be repaired and the mutation patterns they are linked to. **(B)** The percentages of mutations located in RGYW/WRCY and WA/TW motives in HD. All bar plots represent the mean with SD of the age range. **(C)** The percentage of mutations at A/T or G/C locations (divided in transitions and transversions) in all mutations or **(D)** in the total number of base pairs sequenced. **(E)** Sunburst plots showing the substitution patterns in HD. The inner circle represents the original base, while the outer circle shows the newly incorporated base. The figures show the median substitution patterns of all sequenced HD. BER, base excision repair; MMR, mismatch repair.

Somatic hypermutation is initiated by deamination of a C to a U by AID, resulting in a U:G mismatch (Figure 2A). AID has been shown to preferentially target DNA at RGYW and WRCY motifs. In both IGG and IGA transcripts, approximately 35% of the mutations were located in RGYW/WRCY motifs (Figure 2B), and although the variation is small, the frequency negatively correlates with age (Supplementary Figure 1A in Data Sheet 1).

Dependent on the DNA repair pathway used to solve U:G mismatches, transversion and transition mutations at G/C and A/T locations are introduced (Figure 2A). In the HD, more than half (52.8–62.6%) of the mutations occurred at G/C locations (Figures 2C,E, Supplementary Figure 1B in Data Sheet 1), and approximately 50% (45–59.2%) of the mutations at G/C locations were transitions (Figures 2C,E; Supplementary Figure 1B in Data Sheet 1). Mutations at A/T locations are introduced *via* the MMR pathway in which polη has been shown to preferably mutate TW and WA motifs. The frequency and absolute number of mutations at A/T locations (Figures 2C–E), as well as the percentage of mutations in WA/TW motifs slightly correlated with age (Figure 2A; Supplementary Figure 1C in Data Sheet 1). Overall, the repair of the U:G mismatches was very consistent in all HD, except for the cord blood samples in which the targeting and repair patterns were different. This might be related to the very low number of mutations present in these samples and the high level of clonal relation within these samples.

### CDR3 Characteristics Are Different in the Antigen-Experienced Repertoire

**Figure 3:**
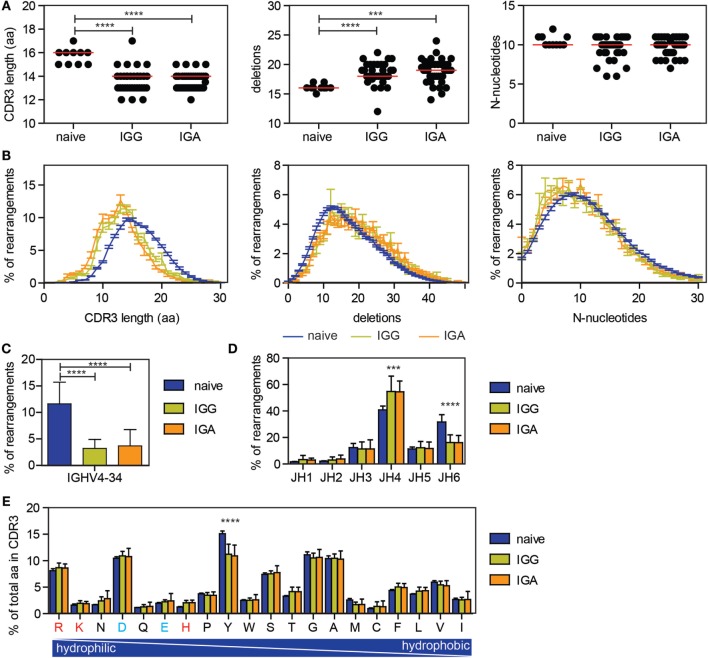
**Differences in CDR3 characteristics and IGHV and IGHJ usage between naive and antigen-experienced repertoire**. **(A,B)** The CDR3 length is reduced upon antigen selection, while the number of deletions is increased and the number of N-nucleotides is comparable between naive and antigen-experienced repertoire. In the dot plots, each dot represents the median of one HD, while the red line indicates the median of all HD. In the line graph, the error bars represents the SE. **(C)** The percentage of transcripts using IGHV4-34 is reduced in the antigen-experienced repertoire. **(D)** JH6 usage is reduced in the antigen-experienced repertoire, while JH4 is more frequently used. **(E)** Tyrosine is less frequently used in the CDR3 of the antigen-experienced repertoire compared with the naive repertoire. Positively charged amino acids are indicated in red and negatively charged amino acids are depicted in blue. In all bar graphs, error bars indicate the SD. Significance was calculated using a one-way ANOVA with a Bonferoni *post hoc* test. *P*-values <0.001 are indicated by *** and *P*-values <0.0001 are indicated by ****.

It has been previously described that antigen-experienced B cells are selected against long CDR3 regions and IGHV4-34 usage, qualities associated with autoimmunity (37). To look at the effect of selection on the CDR3, our data were compared with our previously published next generation sequencing data on productive IGH rearrangements amplified from sorted naive B cells (20). As previously described, a reduced CDR3 length was found in the antigen-experienced repertoire, as well as an increased number of deletions in the junctions (Figures 3A,B). The number of N-nucleotides was similar between the naive and antigen-experienced repertoire (Figures 3A,B).

As previously shown, IGHV4-34 gene usage is significantly lower in the IGG and IGA transcripts compared to naive B cells (Figure 3C) (37). In addition, an increase in IGHJ4 gene usage was found in the antigen-experienced repertoire, while IGHJ6 gene usage was reduced (Figure 3D) (24). Looking at the amino acid usage in the CDR3, the tyrosine (Y) usage was reduced in the antigen-selected repertoire (Figure 3E). These changes were found in all 38 HD and both in IGG and IGA transcripts, indicating that selection for those parameters are uniform and irrespective of age.

### Selection of the Antigen-Experienced Repertoire

**Figure 4:**
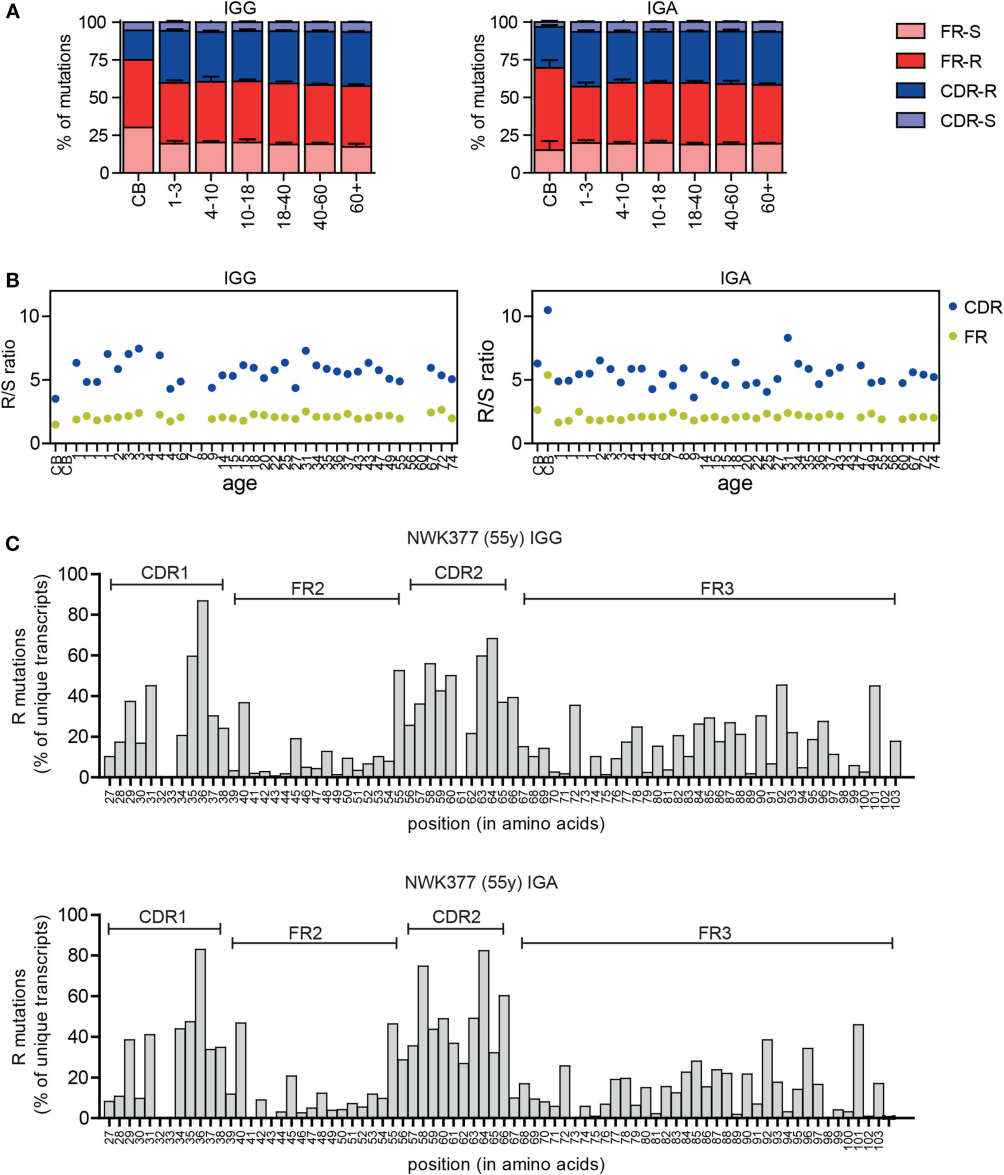
**Replacement and silent mutations in IGG and IGA transcripts**. **(A)** The percentage of replacement (R) and silent (S) mutations in the FR and CDR regions is stable between different age groups except for the cord blood (CB) samples. Means and SDs of the age groups are plotted. **(B)** The R/S ratio in the CDR and FR regions in HD does not correlate with age. **(C)** The frequency of R mutations at different amino acid locations is increased in the CDR regions in IGG and IGA transcripts. A representative sample is shown.

In addition to the changes in the CDR3, selection of the antigen-experienced repertoire can also be measured by analyzing the replacement (R) and silent (S) mutations in the VH gene. B cells with R mutations in the CDRs are positively selected if they increase BR affinity, and B cells with R mutations in the FR regions are negatively selected due to decreased stability of the BR. S mutations do not alter the amino acid sequence and therefore play a limited role in selection. In the IGG and IGA transcripts, the majority of mutations were present in the FR regions (Figure 4A), a consequence of its larger size. Therefore, often the R/S ratio is used to look at antigen selection, as this compensates for the difference in size of the CDR and FR region and for a possible difference in target motives between these regions. In line with selection, the R/S ratio is higher in the CDR compared to FR regions in the HD (Figure 4B). The R/S ratio in the CDR regions was more variable (3.6–8.3), compared to the FR regions (1.6–2.7), but was not correlated to age. In cord blood samples, the R/S ratios were altered. This can most likely be explained by the low mutation rate in these samples and the high clonal relation between transcripts in these samples. Looking at the number of R mutations at the different amino acid locations also confirms an increased frequency of R mutations in the CDR regions (Figure 4C).

**Figure 5:**
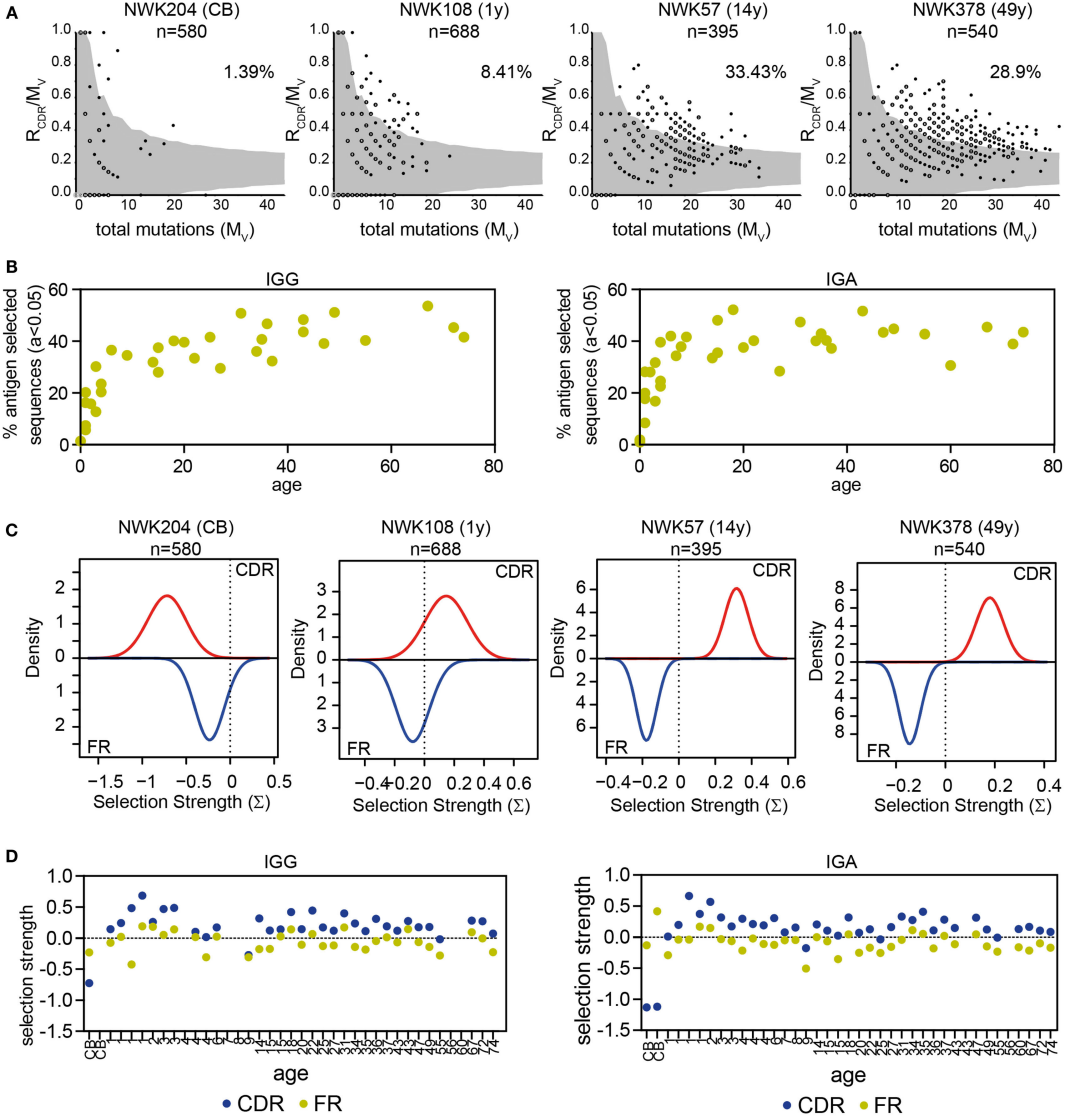
**Antigen selection of IGG and IGA transcripts**. **(A)** Graphical representation of antigen selection in IGG transcripts of four HD as analyzed using IgAT. The ratio of replacement mutations in the CDR regions (R_CDR_) to the total number of mutations (M_v_) is plotted against the M_v_. The gray area represents the 95% confidence interval for antigen selection. All sequences outside of the gray area have undergone antigen selection. The percentage of sequences that have undergone antigen selection is shown in the top right corner. Closed dots represent a single sequence, while open dots represent multiple sequences (as indicated with a number). **(B)** The percentage of antigen-selected transcripts (*a* < 0.05) as analyzed using IgAT increases in early childhood but stabilizes around 6 years of age. **(C)** Graphical representation of antigen selection in four HD as analyzed using BASELINe. **(D)** Quantification of the selection strength as analyzed by BASELINe of the CDR and FR regions in HD of different ages. No correlation is found with age.

In addition to the R/S ratio, several tools have been developed to analyze selection of the repertoire. IgAT looks at the number of R mutations in the CDR in relation to the total number of mutations and uses this to calculate, for each sequence, whether it has undergone antigen selection (31). In this definition of antigen selection, selection is dependent on the number and the location of R mutations but also the frequency of mutations. Applying IgAT to our data showed that the frequency of antigen-selected sequences increased in young children. From 6 years of age onward, the frequency of antigen-selected sequences was around 40% in both IGG and IGA transcripts (Figures 5A,B). The Bayesian estimation of antigen-driven SELectIoN (BASELINe) program uses a similar approach but compares for each transcript the expected mutations based on random distribution of the observed mutations. Subsequently, it calculates the total selection strength for the CDR and FR regions (32, 33). In HD, the selection strength for both the CDR and FR region showed quite some variation; however, the selection strength was always higher in the CDR region, indicating selection (Figures 5C,D).

### Subclass Distribution Changes with Age

**Figure 6:**
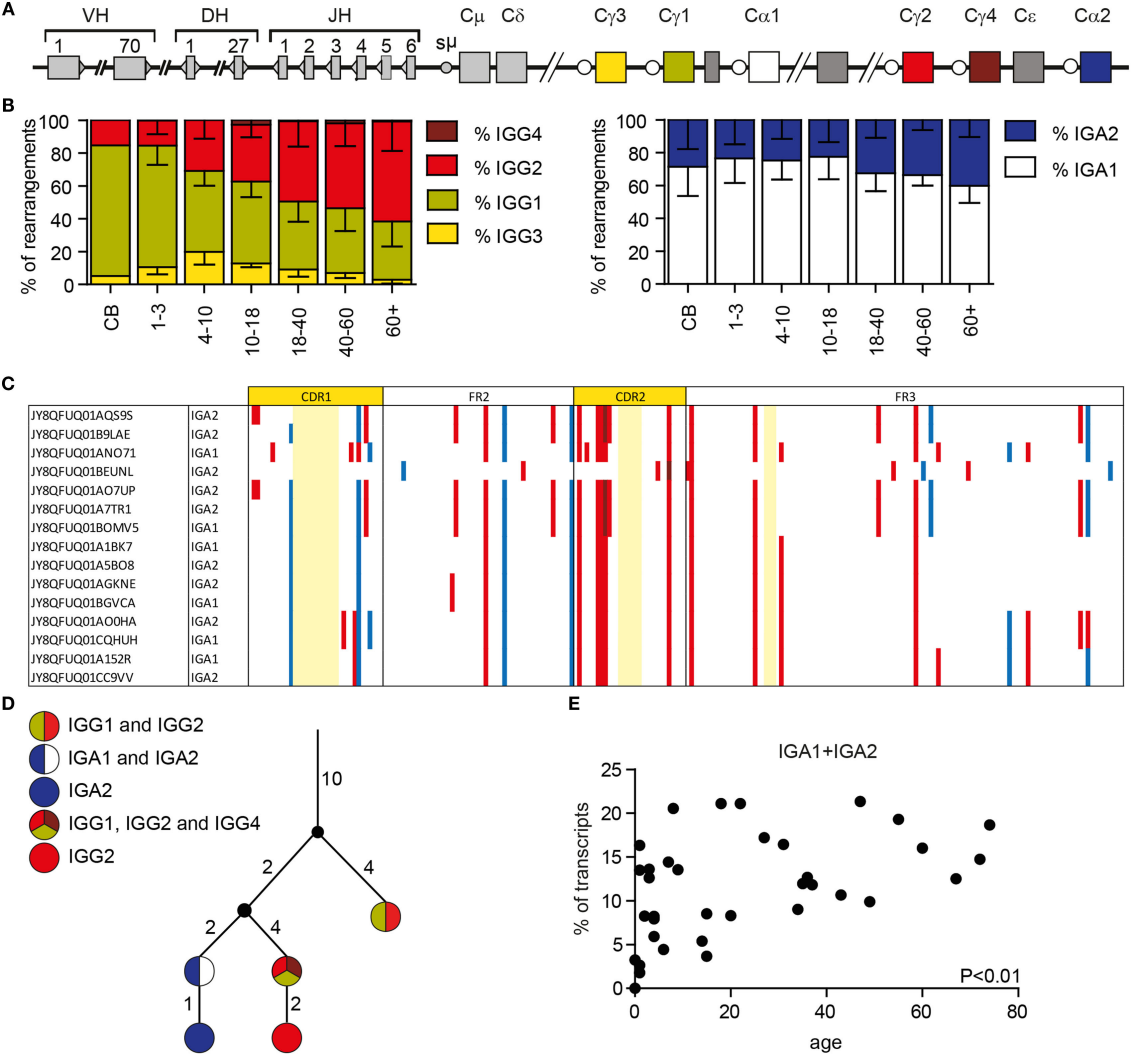

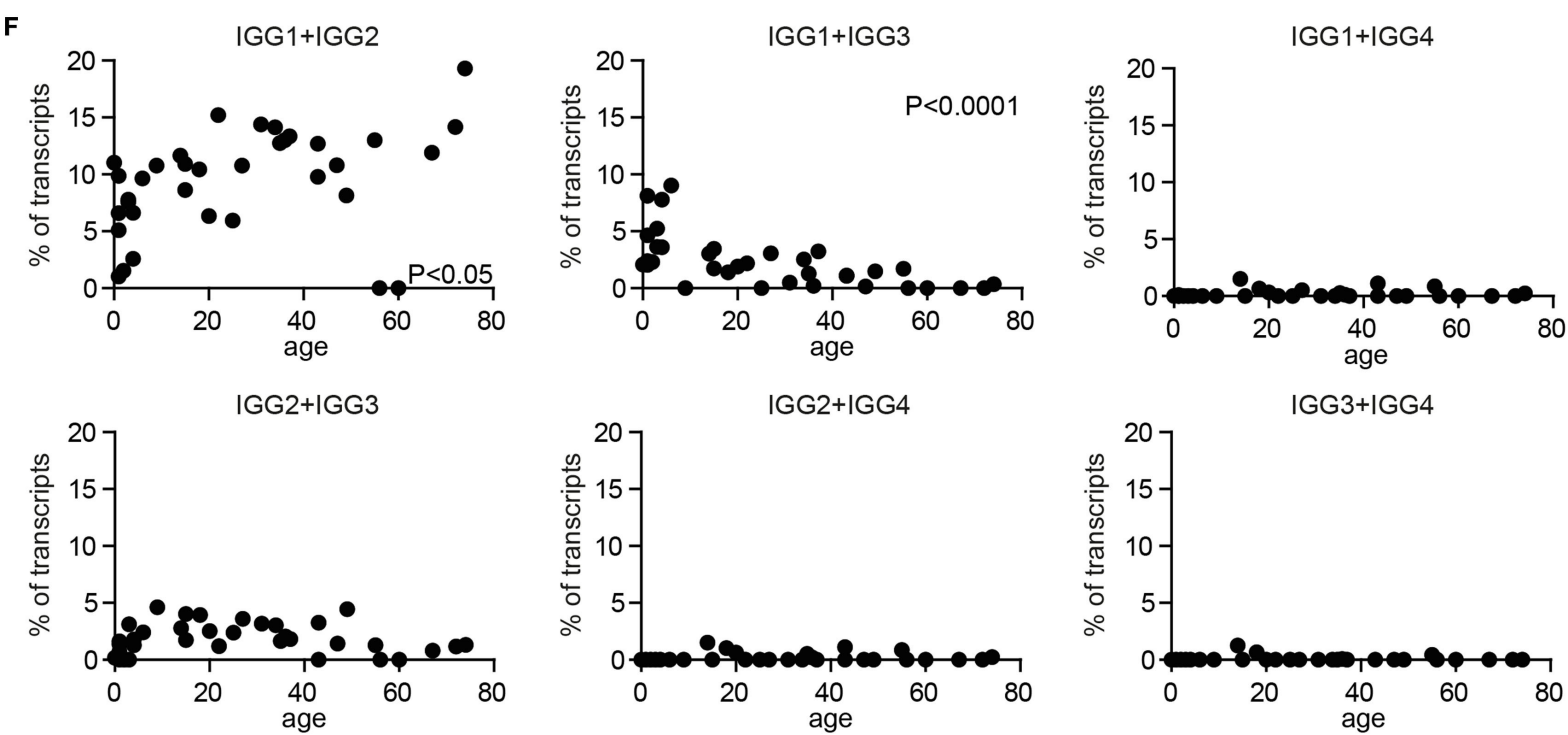
**Class-switching and clonal relationship in HD**. **(A)** A schematic overview of the IGH locus. **(B)** The subclass distribution of IGG and IGA transcripts changes with age. **(C)** Visualization of silent (blue) and replacement (red) mutations in clonal-related sequences. Numbering is based on the IMGT ontology. Bases not present in the clone are marked light yellow. **(D)** Lineage tree of clonal-related sequences found in a single sample based on a minimal substitution model. The small black circles represent inferred sequences not found in our data set. The numbers next to the branches correspond to the number of mutations that occurred between the different sequences. **(E)** The percentage of IGA sequences with the exact same CDR1-CDR3 sequences that are found as both IGA1 and IGA2 transcripts correlate with age. **(F)** The percentage of IGG sequences that overlap between the different IGG subclasses in HD of different ages. *P*-values are calculated using a Pearson correlation test.

Besides SHM, AID also initiates CSR by inducing U:G mismatches in switch regions upstream of the constant genes. The IGH locus contains nine constant genes: Cμ, Cδ, Cγ3, Cγ1, Cα1, Cγ2, Cγ4, Cε, and Cα2 (Figure 6A). We chose reverse primers in the constant domain of the IGG and IGA locus, which allowed us to distinguish between IGA1, IGA2, IGG1, IGG2, IGG3, and IGG4 transcripts. Analysis showed that the subclass distribution of both IGA and IGG is variable between all HD (Supplementary Figure 2 in Data Sheet 1). Looking at IGG transcripts in different age categories showed that the relative amount of IGG2 transcripts is higher with an increased age, while the number of IGG1 transcripts is lower (Figure 6B). The percentage of IGG3 transcripts is low in all HD, except for children from 4 to 10 years of age in which 19.9 ± 7.7% of the IGG transcripts is IGG3. IGG4 transcripts are rare in all different age categories. In the IGA transcripts, the contribution of IGA2 transcripts is increased in samples with higher ages (Figure 6B).

### Clonal Relation

Each antibody subclass has a different effector function; however; it is still not completely understood how class switching occurs during an immune response. We used Change-O to identify clonally related sequences within our data set that likely originate from a single precursor B cell (34). For each sample, we have calculated the maximal clone size (the maximal percentage of transcripts that are part of one clone), and the percentage of sequences from unique clones (Supplementary Table 1 in Data Sheet 2). These data represent the clonality of the analyzed data but are not necessarily representative of the clonality of the donor repertoire. We identified several clones in every HD (Supplementary Table 1 in Data Sheet 2). These clones differ in size, and the number of acquired mutations. Assigning clonal relation allows comparison of SHM patterns within a single clone (Figure 6C), and the creation of lineage trees of clonal outgrowth (Figure 6D). Interestingly, we found multiple clones that contained transcripts of different subclasses within the same class (IGA or IGG) (Figure 6C) or both IGA and IGG transcripts (Figure 6D). This shows that within one antigen-driven response, CSR to multiple subclasses can be found. Looking into this further, we also identified sequences with exactly the same CDR1–CDR3 sequence, and thus the same SHM pattern, but different constant genes. The frequency of IGA1 and IGA2 transcripts with exactly the same CDR1–CDR3 increased with age (Figure 6E). In addition, we found more overlap between IGG1 and IGG2 sequences with increased age, while the overlap between IGG1 and IGG3 is deceased with increased age (Figure 6F). This might be at least partly related to the altered subclass distribution within different age categories. We also found sequence overlap between IGA and IGG sequences as summarized in Supplementary Table 3 in Data Sheet 2. The presence of these transcripts suggests that, during the course of an immune response, some B cells change their isotype without acquiring additional mutations, or B cells can directly switch to different subclasses.

## Discussion

In this study, we analyzed IGG and IGA transcripts from class-switched B cells from 38 HD to study SHM and CSR from neonates to elderly (0–74 years). The raw data set of this study contains 412,890 sequences of IGG and IGA transcripts and 781,022 genomic sequences of naive B cells, which will be freely available via the ENA website (PRJEB15348) This will allow our data to be used as reference data for the naive and antigen-experienced B cell repertoire in HD of different ages. We believe that this data set can be highly valuable for repertoire studies in different fields of research such as primary immunodeficiency, lymphoma, or autoimmunity. In addition, this type of data can benefit the field of bioinformatics in which freely available data sets are often used for testing and developing novel analysis tools.

Because of our interest in SHM, we needed to distinguish between sequencing errors and true SHM in our study. Therefore, we only included unique transcripts of which the exact CDR1–CDR3 nucleotide sequence occurred twice or more. This strategy described by Yaari et al. (32) strongly reduces the sequencing errors in an unbiased way and thereby improved the reliability of the data. A disadvantage of this approach is that it results in a great loss in the number of transcripts. This could be a problem for specific studies in which limited numbers of transcripts are available. In these cases, a different algorithm for sequencing error removal is needed. Ig-Indel-Identifier is a newly developed tool, which provides a way to exclude sequencing errors from BR repertoire data without losing the majority of sequences (38).

Until now, SHM and CSR patterns in healthy children and adults have only been addressed in studies with limited number of sequences or small cohorts (21–23). Similar to previous studies (21–23), we found that the frequency of mutations was low in cord blood and increased in young children. In contrast to the study of Schatorje et al. in which they showed a rapid increase of mutations in the first 2 years of life, we observed that the frequency of mutations is not stable until around 6 years of age. These differences likely result from the difference in chains analyzed (IGKV vs. IGHV) and the different assays used for determining the mutation frequency. The mutation-hot spot assay used by Schatorje et al. gives an estimation of the mutation frequency by determining the percentage of expressed IGKV3-20 genes that have a mutation in a specific hot spot motif in the total B-cell population, while our study analyzes the mutation frequency in IGH transcripts isolated from class-switched B cells. The increasing mutation frequency up to 6 years of age, probably, reflects the accumulation of successive rounds of SHM.

In our study, the targeting and patterns of SHM mutation were quite consistent between all HD. Even though the variations between the different age groups were small, we did observe a correlation with age for the frequency of mutations in the AID targeting motifs (RGYW/WRCY), polη motifs (WA/TW), and the frequency of mutations at A/T mutations in both IGG and IGA transcripts. This is not linked to the frequency of mutations as there is no difference in SHM targeting and patterns between the age groups 1–3 and 4–10, while the difference in mutation frequencies is the greatest. In the cord blood samples, the SHM targeting and mutations deviate from the other HD. This might be the result of the very low mutation frequency (0–0.5%) in these samples, which results in low number of mutations to be analyzed. In addition, both cord blood samples have many clonally related sequences, which could heavily bias results.

After SHM, which takes place in the dark zone of the germinal center, B cells migrate to the light zone of the germinal center. Here, selection based on the affinity of the BR takes place; cells with reduced affinity undergo apoptosis, while cells with increased affinity are selected for survival (39). B cells with affinity increasing mutations in the CDR regions are selected for survival, while there is selection against R mutations in the FR regions. S mutations do not alter the amino acid sequence and, therefore, their effect on affinity and selection is limited. Here, we analyzed antigen selection using different described methods.

The R/S ratio’s and BASELINe analysis showed more R mutations in the CDR, compared to the FR regions, confirming selection for those mutations. Interestingly, the R/S ratio and selection strength is stable between all HC, suggesting that antigen selection is relatively stable with age. In contrast, analysis using IgAT suggests that selection is increased with age in early childhood and stabilizes at around 6 years of age (31). This discrepancy can be explained by the difference in calculation methods for antigen selection between the three tools. In the calculation of the R/S ratio’s, the amount of antigen selection is based on the number and location of R and S mutations in all transcripts. IgAT and BASELINe, both calculate the amount of antigen selection per transcript by looking at the ratio of R mutation in the CDR region in each individual transcript. Subsequently, BASELINe includes all transcripts to calculate the selection strength, while IgAT only assigns transcripts as antigen-selected above a certain threshold. The latter approach is skewed against antigen selection in sequences with low levels of SHM explaining the limited amount of antigen selection in early childhood found when using IgAT. At the moment, a real consensus on the definition of antigen selection is missing. A way to further examine this would be to combine repertoire sequencing of antigen-specific B cells and linking these to affinity measurements of the sequenced BR. However, large data sets from different antigen-specific responses are needed to develop and test a novel algorithm. Novel techniques allowing linked sequencing of the heavy and light chains from a single cell will allow high-throughput production of this type of data and, hopefully, provide more insight on the best definition of antigen selection.

Three different models have been proposed for class switching; direct switching to downstream subclasses and indirect switching via the temporal model or the sequential model (40–43). In the temporal model, sequential switching from IgG3 > IgG1 > IgG2 > IgG4 occurs during the course of an immune response, while in the sequential model, a second round of antigen exposure leads the IgG^+^ B cells to re-enter the germinal center and undergo class switching to more downstream constant genes. The increase in IGG2 transcripts and the decrease of IGG1 transcripts with age found in this study match the sequential model. With age, the chance of encountering antigens multiple times increases, allowing IgG1^+^ B cells to undergo class switching to more downstream constant genes such as IGG2. Our finding of clonally related or the same sequences with different subclasses could point toward simultaneous class switching to multiple subclasses. This would be more in line with the direct class switching model. It seems most likely that *in vivo* both or even all three models occur in parallel. This is further supported by findings in the study described by Berkowska et al. in which they analyzed the switch regions of IGG2 rearrangements and found that around 76% of the cells switch directly to IGG2, while 24% of the cells had undergone indirect switching via IGG1, IGG3, or IGA1 (43). The presence of clonally related sequences with a different subclass further suggests that BR of different subclasses can be formed against a single antigen.

In summary, here we present a large high-throughput sequencing data set of IGG and IGA transcripts of 38 HD of different ages (0–74 years). Our study showed that the level of mutations are increasing with age in early childhood, and the frequency of IGG2 and IGA2 transcripts correlated with age, emphasizing the importance of using age-matched controls when analyzing the BR repertoire. Moreover, we identified IGH clones derived from an ancestry B cell, which acquired additional mutations. In addition, we found IGH rearrangements with exactly the same SHM pattern but with different subclasses, giving new insights in the development of the immune repertoire. This analysis opens new opportunities for studying immune maturation in different patient groups.

## Author Contributions

HI, PS, and MB designed research; HI, PS, and MB wrote the manuscript; HI, PS, and IP-K performed experiments. DZ, AS, HI, and PS developed an analysis pipeline and analyzed the data. GD contributed to essential discussion of the paper and critically read the manuscript.

## Conflict of Interest Statement

The authors declare that the research was conducted in the absence of any commercial or financial relationships that could be construed as a potential conflict of interest.

## Footnotes

^1^http://454.com/downloads/USM-00058.09_454SeqSys_SWManual-v2.9_PartB.pdf

^2^http://454.com/downloads/my454/documentation/gs-junior/software-manual/454_Sequencing_Software_Manual_v2.5p1_PartB.pdf
