# Supplementary material for: Evaluation of the Antigen-Experienced B-Cell Receptor Repertoire in Healthy Children and Adults
Source: Front Immunol. 2016 Oct 17;7:410. doi: 10.3389/fimmu.2016.00410 (PMC5066086; doi:10.3389/fimmu.2016.00410)
Supplement: Supplementary file 1 [file Data_Sheet_1.DOCX]

Supplementary Material

**Development of antigen-experienced B-cell receptor repertoire in healthy children and adults**

Hanna IJspeert,^#^ Pauline A. van Schouwenburg,^#^ David van Zessen, Ingrid Pico-Knijnenburg, Gertjan J. Driessen, Andrew P. Stubbs, *Mirjam van der Burg

*** Correspondence:** Corresponding Author: [m.vanderburg@erasmusmc.nl](mailto:m.vanderburg@erasmusmc.nl)

# Supplementary Figures and Tables

**
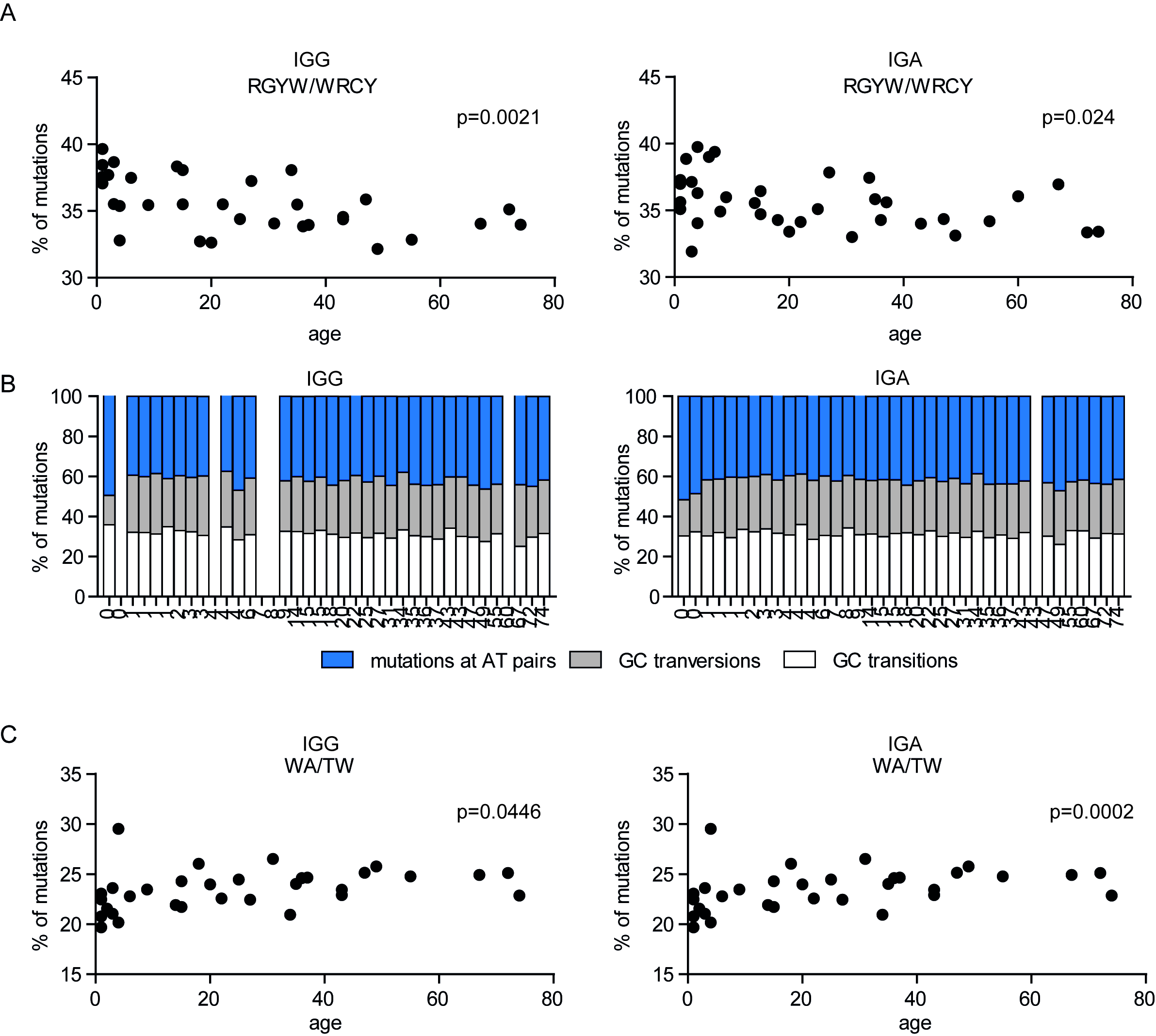
**

**Supplementary figure 1. Targeting and substitution patterns of SHM in HD in IGG and IGA transcripts. (A)** The small variation in the percentage of mutations located in RGYW/WRCY motives negatively correlates with age. **(B)** The percentage of SHM in A/T locations or G/C locations (divided in transitions and transversions) for each individual control. There is a positive correlation between age and mutations at A/T locations (IGG: r=0.48, p<0.01; IGA: r=0.53, p<0.001). In IGG transcripts the number of transitions at GC locations decreases with age (r=-0.48, p<0.0056), while in IGA there is no significant correlation (r=-0.21, p=0.23). In IGA transcripts the number of transversions at GC locations is negatively correlated with age (r=-0.39, p<0.05), while no correlation was found in IGG transcripts (r=-0.10, p=0.58). **(C)** The percentage of mutations in WA/TW motives slightly increases with age.


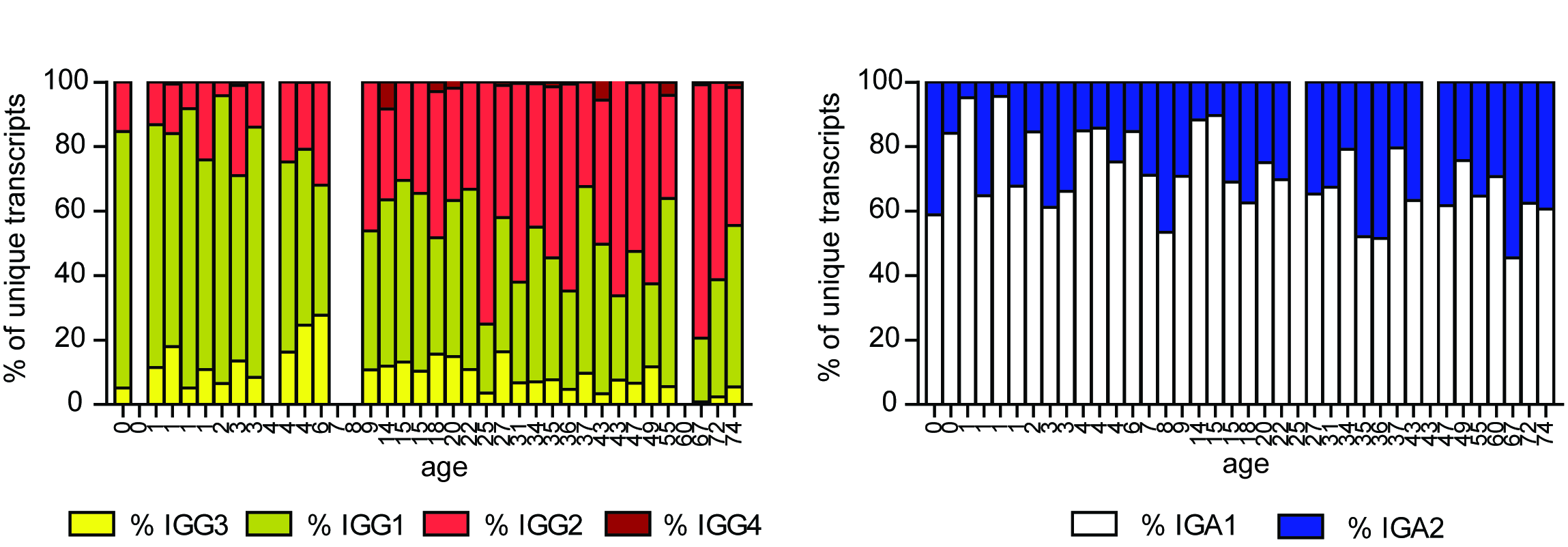


**Supplemental Figure 2. IGG and IGA subclass distribution.** The IGG and IGA subclass distribution varies per individual, but overall the percentage of IGG2 and IGA2 transcripts increases with age.
